# Editorial: Novel Multimodal Approaches in Non-invasive Brain Stimulation

**DOI:** 10.3389/fnhum.2021.784637

**Published:** 2021-11-19

**Authors:** Nivethida Thirugnanasambandam, Florian H. Kasten, Kaviraja Udupa

**Affiliations:** ^1^Human Motor Neurophysiology and Neuromodulation Lab, National Brain Research Centre (NBRC), Manesar, India; ^2^Experimental Psychology Lab, Department of Psychology, European Medical School, Cluster of Excellence “Hearing4All”, Carl von Ossietzky University, Oldenburg, Germany; ^3^Department of Neurophysiology, National Institute of Mental Health and Neurosciences (NIMHANS), Bengaluru, India

**Keywords:** brain stimulation, multimodal, tACS-EEG, repetitive transcranial magnetic stimulation (rTMS), transcranial electrical stimulation (tES)

Over the last two decades, the number of techniques that fall into the realm of non-invasive brain stimulation (NIBS) has increased due to their immense potential in the diagnosis and treatment of neuropsychiatric diseases. Further, researchers have successfully integrated some of these techniques with neurophysiological/neuroimaging methods with the aim of enriching our understanding of brain function and of mechanisms underlying the effects of stimulation on the brain. However, such a multimodal approach is not without challenges and pitfalls. This Research Topic brings together original articles, reviews and a perspective article showcasing advances in multimodal NIBS approaches for studying physiological and pathological states of the brain.

All original research articles were based on work done using low intensity transcranial electrical stimulation (tES). The first one is an elaborate study by Karabanov et al. that used fMRI to probe the after-effects of transcranial direct current stimulation (tDCS) on motor cortical excitability, visuomotor task performance and task-related neural activity. The study revealed inconsistent group effect on primary outcome measures owing to high response variability. However, the authors report significant changes in the supplementary motor area, upstream from the primary motor cortex. Their findings warrant a more detailed data-driven approach for analyzing brain stimulation data. The four research articles on combination of transcranial alternating current stimulation (tACS) and EEG show the strong interest on this topic. On the one hand, Zarubin et al. and Stecher et al. have clearly demonstrated the inconsistencies observed in closed loop tACS-EEG studies which could probably be due to the subthreshold stimulation intensity of tACS being too low to bring about a consistent effect and/or alternative mechanisms other than entrainment that may underlie the effects of tACS. While Zarubin et al. showed that modulation of intrinsic oscillations by tACS is independent of the phase relationship between the signals, Stecher et al., demonstrated that matching the frequency of the tACS signal to that of the intrinsic neural signal using the closed-loop setup did not modulate behavior, rather the fixed-frequency tACS did. These results necessitate further research on the mechanisms of NIBS effects and factors that impact behavioral and neurophysiological modulation. On the other hand, two studies aimed to address the technical challenges in data acquisition and analysis using tACS-EEG—Vosskuhl et al. tested the feasibility of applying signal-space-projection to reduce tACS-induced artifacts in the EEG and compared its performance to other approaches such as sine and template subtraction. Holzmann et al. describe the removal of tACS artifacts with a special emphasis on non-linear amplitude modulations of the artifact, which occur due to physiological processes in the body such as heartbeat or respiration, using a multi-step artifact removal procedure, which they assess using a novel demonstrator setup, that allows to simulate these non-linear dynamics.

Besides original studies, the Research Topic features four review articles discussing general theoretical considerations in the combination of NIBS and neuroimaging as well as their application in clinical settings. The article by Hobot et al. is timely and discusses the limitations of making causal inferences in repetitive transcranial magnetic stimulation (rTMS) research. The authors have critically evaluated literature for studies that have inappropriately used TMS methods to answer causality-related questions and have emphasized on the importance of designing experiments carefully to draw such inferences. In addition, three review articles extensively cover the clinical and research implications of multimodal NIBS in specific pathological states namely schizophrenia, movement disorders and depression. While Shukla and Thirugnanasambandam showcase the untapped potential of multimodal NIBS in understanding the pathophysiology of movement disorders and call for multidisciplinary collaborations for better progress in the field, Baliga and Mehta have focused on the numerous studies that shed light on the pathophysiological mechanisms and scope for personalized treatment in schizophrenia by combining TMS with functional magnetic resonance imaging (fMRI). Finally, Sinha et al. have presented a systematic review and coordinate-based meta-analysis of the effects of electroconvulsive therapy (ECT) on the resting state functional connectivity in depression.

A major highlight under this Research Topic is the perspective article in which Ruhnau and Zaehle propose a novel combination of transcranial auricular vagus nerve stimulation (taVNS) and ear-EEG in a closed loop fashion that could have the potential to modulate attention. The authors have discussed how ear-EEG, a relatively novel form of mobile EEG technique, can reliably record alpha activity from the parietal and temporal brain regions, and when fed in to stimulate the vagus nerve non-invasively in a closed loop manner could modulate attention.

Overall, this Research Topic nicely portrays the spectrum of research undertaken using multimodal NIBS spanning from pathophysiology to clinical applications and from technical advances to behavioral impact. We can conclude that there is a unanimous agreement among the scientific community on the enormous potential of multimodal NIBS approaches to largely improve our understanding of brain function. However, currently there are several limitations that need to be addressed, especially to obtain more consistent and reproducible responses. Although we have made great progress over the past decade in exploring the vast prospects of multimodal NIBS, a more collaborative and multi-disciplinary approach will be necessary to confront technical challenges and optimize protocols for clinical and research applications. The collection of articles in this Research Topic have portrayed this convincingly.

## Author Contributions

All authors listed have made a substantial, direct, and intellectual contribution to the work and approved it for publication.

## Conflict of Interest

The authors declare that the research was conducted in the absence of any commercial or financial relationships that could be construed as a potential conflict of interest.

## Publisher's Note

All claims expressed in this article are solely those of the authors and do not necessarily represent those of their affiliated organizations, or those of the publisher, the editors and the reviewers. Any product that may be evaluated in this article, or claim that may be made by its manufacturer, is not guaranteed or endorsed by the publisher.

